# Coagulation parameters as a guide for fresh frozen plasma transfusion practice: A tertiary hospital experience

**DOI:** 10.4103/0973-6247.59387

**Published:** 2010-01

**Authors:** W. M. Wan Haslindawani, A. Wan Zaidah

**Affiliations:** *Department of Hematology, Health Campus, Universiti Sains of Malaysia, Kubang Kerian, Kelantan, Malaysia*

**Keywords:** Coagulation, transfusion, fresh frozen plasma, rational use of plasma

## Abstract

**Introduction:**

The appropriate use of blood and blood products means the transfusion of safe blood products only to treat a condition leading to significant morbidity or mortality, which cannot be prevented or managed effectively by other means. The safety and effectiveness of transfusion depend on the appropriate clinical use of blood and blood products. This study was conducted to review the practice of fresh frozen plasma usage (FFP) for transfusion, based on the coagulation profile, requested by various departments in the Hospital Universiti Sains Malaysia (HUSM).

**Methodology:**

A retrospective review of blood bank records and coagulation profile results of the patients given FFP from October to December 2006, in Hospital USM was undertaken. The criteria set by the College of American Pathologists in 1994, were used as the guidelines.

**Results:**

One thousand six hundred and ninety-eight units of FFP were used during this study period. Only 806 (47.47%) FFP units were deemed appropriate. 20.38% were based on studies without any coagulation tests prior to transfusion and 21.13% were transfused for mild prolongation of coagulation test results. About 6.41% requested FFP in the setting of normal coagulation results.

**Conclusion:**

Our results showed that a significant proportion of the FFP transfusion was not guided by the coagulation profile. We recommend that a continuous education on FFP transfusion may help to guide the appropriate request for FFP.

## Introduction

Transfusion carries the risk of adverse reactions such as allergic reactions, anaphylaxis, transfusion-related lung injury, and hemolysis from transfused antibodies to blood group antigens, especially A and B.[1] Plasma can transmit most of the infections present in whole blood. Fresh frozen plasma (FFP) is a blood product produced from plasma that is separated from packed red cells and platelets, after centrifugation of donated whole blood, and frozen to −30°C or below within six hours after collection.[2] It is a good source of coagulation factors, including labile factors V and VIII.

The indications for transfusing FFP, cryo precipitate, and cryosupernatant plasma are very limited. Guidelines for the use of FFP have been developed by several expert groups. The “Practice Parameters for the use of FFP” by the Task Force of the College of American Pathologists in 1994, was used in this retrospective study, to provide the basis for the appropriate use of FFP [Table 1].[3]

**Table 1:** Fresh frozen plasma usage transfusion guidelines, college of american pahtologists, 1994

Department/unit	Units of fresh frozen plasma	Percentage of frozen plasma fresh frozen plasma usage
General surgery	158	9.30
General medicine	143	8.42
Neurosurgical	262	15.43
Orthopedics	33	1.94
Intensive care	545	32.10
Cardiac care	48	2.83
Ear, nose, and throat	20	1.18
Accident and emergency	73	4.30
Operation theater	56	3.30
Burn	42	2.47
Obstetrics and gynecology	55	3.24
Oncology	120	7.07
Others	12	0.71
Total	1698	

There are few indications for FFP usage, which will be similar to the replacement of multiple coagulation factor deficiencies, such as, in liver disease, warfarin overdose, or depletion of coagulation factors in patients receiving large volume transfusions. Disseminated intravascular coagulation (DIC) and thrombotic thrombocytopenic purpura (TTP) will be other indications. In certain cases where there is a history or clinical course suggestive of coagulopathy, it must be documented by at least one of the following to justify administration of FFP — PT more than 1.5 times the midpoint of normal range, aPTT more than 1.5 times the top of the normal range, or coagulation assay of less than 25% activity.[4]

Our hospital is a teaching hospital as also a referral center for various disciplines in the East Coast of Malaysia, such as, Neurosurgical, Cardiothoracic, Oncology (adult and Pediatric), Solid tumor (Orthopedics), as well as burn cases. We have noted that the usage for FFP in our hospital is quite high (more than half the number of units of red cells transfused each month). This study presents an analysis of the practice of FFP transfusion in our hospital as one of the teaching hospitals and tertiary referral centers, with the availability of expertise and facilities in various specialties.

## Materials and Methods

This retrospective study was conducted from October until December 2006. Blood bank records and coagulation profile results of the patients given FFP from October to December 2006, in Hospital USM, were taken. Data such as department requesting for FFP, patient's presenting problem, reason for FFP request, date of transfusion, number of units transfused, coagulation profile of patient, and causes of coagulopathy if investigated, were recorded. The criteria set by the College of American Pathologists in 1994, were used as guidelines [Table 1]. An FFP transfusion was considered inappropriate if (i) a coagulation profile was not done at the time of request and (ii) the prolongation of PT/PTT was less than 1.5 times that of normal control plasma.

## Results

During the study period, 1698 units of FFP were used, and only 806 (47.47%) units were appropriate. This means that 892 units of FFP were transfused inappropriately. A majority of inappropriate transfusions were not accompanied by coagulation test results as a guide to the transfusion and for monitoring purposes. Some of the cases were not justified to be transfused (normal coagulation profile).

**Figure 1:**
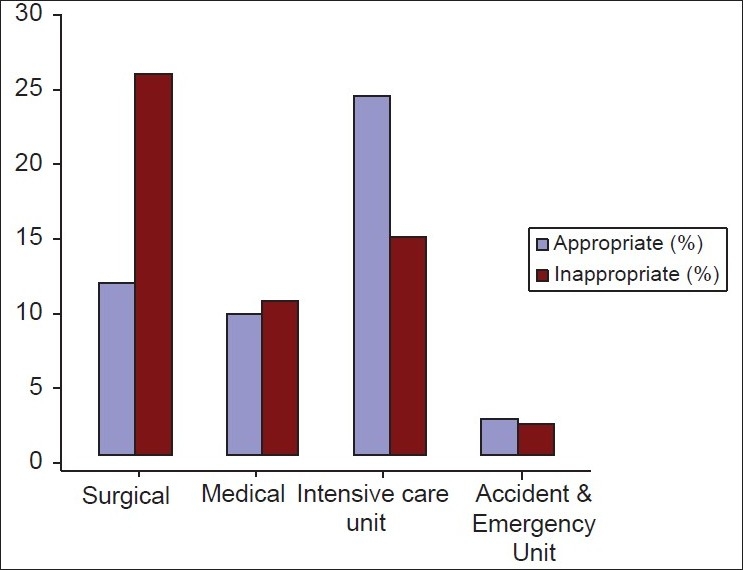
Appropriate vs inappropriate usage of fresh frozen plasma request by discipline

Fresh frozen plasma was used by both medical and surgical specialties, with the Neurosurgical Unit (15.43%) and Intensive Care Unit (32.1%) being the main users [Table 2]. Surgical-based departments were the highest discipline that requested for FFP transfusions, without information on the coagulation profile (25.56%). The Intensive Care Unit (ICU), showed that most of the requests were based on the coagulation profile of the patients (24.09 vs. 14.60%) [Figure 1].

**Table 2:** Distribution of fresh frozen plasma usage according to various departments/units

History or clinical course suggestive of a coagulopathy due to congenital or acquired deficiency of coagulation factors, with active bleeding or other invasive procedures. This must be documented by at least one of the following:	
PT greater than 1.5 times the mid point of the normal range aPTT greater than 1.5 times the top of the normal range Coagulation assay of less than 25% activity	
Massive blood transfusion:	Replacement of more than one blood volume within several hours, with evidence of a coagulation deficiency as in (1), with continued bleeding
Reversal of warfarin effect:	If immediate hemostasis is required to stop active bleeding or prior to emergency surgery or an invasive procedure (PT > 18 seconds or INR > 1.6)
Prophylactically for surgery or an invasive procedure in cases of documented congenital or acquired coagulation factor deficiency Deficiency of antithrombin, heparin cofactor 11, protein C or protein S	
Plasma exchange for thrombotic thrombocytopenic purpura or hemolytic uremic syndrome	

Disseminated intravascular coagulopathy (DIC) was the main reason for transfusion, followed by bleeding, mainly in neurosurgical cases. The other reason for FFP transfusion was as a prophylaxis to anticipated bleeding.

Some of the patients with acute massive blood loss (in motor vehicle cases) were given FFP prior to the availability of the results of coagulation screening tests, due to the urgency of the situation.

Three hundred and forty-six units (20.38%) of FFP were transfused without any coagulation profile done to the patients prior to the transfusion. Three hundred and sixty-two units (21.13%) requested FFP for correction of only mild prolongation of coagulation profile. There was a small percentage of FFP requested in the setting of a normal coagulation profile (6.41%), probably given as prophylaxis (as seen in burn cases).

## Discussion

Fresh frozen plasma is a frequently prescribed blood product. Inappropriate use of FFP exposes patients to risk of transfusion transmissible diseases or allergic and hemolytic reactions caused by A and B antibodies. In rare cases, antibodies against the patient's granulocytes may cause leukocyte aggregation in pulmonary vessels leading to transfusion-related lung injury (TRALI syndrome).[5] Any inappropriate use of blood and its components will lead to a wastage of limited resources, depriving more needy patients of their use, increased healthcare cost, and risk of transfusion-related complications, such as viral transmission, which could lead to significant morbidity and mortality. Therefore, it should only be used when there is a documented coagulation defect, which could be corrected by a reasonable amount of FFP.

Despite the availability of guidelines and protocols, a high rate of inappropriate use has been reported around the world, both in the developed and developing countries.[6] The high rates of inappropriate transfusion may reflect the uncertainty among clinicians and surgeons, treating the patients, about the appropriate laboratory criteria that forms the basis for FFP usage for clotting support, which can be exemplified by 21.13% of patients receiving transfusion with PT/PTT values of < 1.5 times the normal range (although it is not justified).

Fresh frozen plasma transfusion prophylactically for patients who do not have abnormal coagulation results before or after procedures, with the potential for hemorrhage, is another area of inappropriate use. This misuse was largely due to misconceptions regarding the hemostatic effectiveness of FFP,[7] although there have been so many efforts to improve these misconceptions by conducting seminars or by continuous medical education to all the surgeons as well as clinicians.

Attempts to formulate guidelines for proper FFP use have been faced with controversy and lack of a firm scientific foundation. However, a program of daily monitoring of FFP usage combined with continuous education has significantly reduced the usage of FFP by 77% at the William Beaumont Hospital.[8] A systematic review of FFP requests may be more educational and beneficial in the long run compared to a retrospective review. Some centers have also modified blood product request forms, to incorporate indications for transfusion, based on clinical and laboratory findings.[9] Here, in our center, in cases where there is no indication stated on the request forms, we follow up with the treating doctors to clarify whether the indications for transfusion are justifiable or not.

Furthermore, investigations of all abnormal APTT/PT are necessary as a guide for appropriate treatment or transfusions. Any use of FFP needs to be followed by a coagulation profile request as a monitoring basis, to assess the adequacy of transfusions, and this will be the guide to whether further transfusions are mandatory or not.

With the exception of emergency situations, when timely clotting assay results are not available, the administration of plasma in coagulopathy is judged mainly for clinical perspectives as has occurred in 20.38% of the cases. In this situation, it is justified to give the products prior to the laboratory parameters becoming available.

There are other situations where products more effective and safer than FFP are available for correction of coagulopathy, such as, recombinant or virally inactivated specific clotting factor concentrates, for treatment of hemophilia, von Willebrand's disease, and hypofibrinogenemic states, and prothrombin complex concentrates and vitamin K for warfarin reversal.[10]

## Conclusion

This study indicates that the practice of FFP transfusion needs to be improved. An FFP request should be based on clinical and laboratory evidence of coagulopathy. In the long-term, there is a need to monitor and review all the requests for FFP continuously, to ensure a safe and cost-effective practice of FFP transfusion.
